# Gene Therapy for Pancreatic Cancer: Specificity, Issues and Hopes

**DOI:** 10.3390/ijms18061231

**Published:** 2017-06-08

**Authors:** Marie Rouanet, Marine Lebrin, Fabian Gross, Barbara Bournet, Pierre Cordelier, Louis Buscail

**Affiliations:** 1 Department of Gastroenterology, CHU Rangueil, 1 avenue Jean Poulhès, Toulouse 31059, France; marie.rouanetvidal@gmail.com (M.R.); bournet.b@chu-toulouse.fr (B.B.); 2 INSERM UMR 1037, Cancer Research Center of Toulouse, Toulouse 31037, France; pierre.cordelier@inserm.fr; 3 Center for Clinical Investigation 1436, Module of Biotherapy, CHU Rangueil, 1 avenue Jean Poulhès, Toulouse Cedex 9, France; lebrin.m@chu-toulouse.fr (M.L.); gross.f@chu-toulouse.fr (F.G.); 4 University of Toulouse III, Medical School of Medicine Rangueil, Toulouse 31062, France

**Keywords:** gene therapy, pancreatic cancer, expression vectors, clinical trial, oncovirus, vaccination, endoscopic ultrasound

## Abstract

A recent death projection has placed pancreatic ductal adenocarcinoma as the second cause of death by cancer in 2030. The prognosis for pancreatic cancer is very poor and there is a great need for new treatments that can change this poor outcome. Developments of therapeutic innovations in combination with conventional chemotherapy are needed urgently. Among innovative treatments the gene therapy offers a promising avenue. The present review gives an overview of the general strategy of gene therapy as well as the limitations and stakes of the different experimental in vivo models, expression vectors (synthetic and viral), molecular tools (interference RNA, genome editing) and therapeutic genes (tumor suppressor genes, antiangiogenic and pro-apoptotic genes, suicide genes). The latest developments in pancreatic carcinoma gene therapy are described including gene-based tumor cell sensitization to chemotherapy, vaccination and adoptive immunotherapy (chimeric antigen receptor T-cells strategy). Nowadays, there is a specific development of oncolytic virus therapies including oncolytic adenoviruses, herpes virus, parvovirus or reovirus. A summary of all published and on-going phase-1 trials is given. Most of them associate gene therapy and chemotherapy or radiochemotherapy. The first results are encouraging for most of the trials but remain to be confirmed in phase 2 trials.

## 1. Introduction

Pancreatic ductal adenocarcinoma (PDAC) is the fifth leading cause of cancer-related death in Western countries, and its incidence has increased over the last 40 years [1]. Unfortunately the prognostic remains poor and a recent death projection of the “top cancer killers” due to demographic changes has placed PDAC as the second cause of death by cancer in 2030 behind the pulmonary carcinoma [2]. Curative surgery to manage PDAC is possible in only a fraction of patients. Indeed, a vast majority (85%) of patients are diagnosed with locally advanced tumors and/or metastases because of lack specific symptoms and early markers for this dismal disease. For these patients, palliative armamentarium consists of conventional chemotherapeutic agents such as gemcitabine and, more recently, FOLFIRINOX (protocol 5-FU, Folinic Acid, Irinotecan, Oxaliplatin) and nab-paclitaxel, which offer marginal survival benefits [3,4,5,6]. Even if promising innovative treatments based on patient genetics (PARP inhibitor), or targeting the tumor microenvironment (hyaluronidase, sonic hedgehog inhibitors), are currently being evaluated in early phase clinical trials, the prognosis for PDAC is still very poor and there is great need for new treatments that can change this poor outcome [7,8,9].

Unlike other digestive-cancer entities, such as colon cancer or gastrointestinal stromal tumors, molecular-targeted therapies have, so far, largely failed to improve patient survival in PDAC [1]. Indeed, large-scale genetic analyses have recently revealed that PDAC is defined by numerous exomic alterations in diverse signalling pathways, which vary considerably between patients. In addition, primary tumors are intrinsically heterogeneous and specific cellular subclones can be identified in metastasis. Such heterogeneity hampers the development of targeted therapies for the intervention in PDAC [9,10,11].

In this context, and in the past few years, several teams and we have developed innovative approaches based on therapeutic gene transfer namely gene therapy. In this review, we will comment on the latest developments in PDAC gene therapy, including gene-based tumor cell sensitization to chemotherapy, tumor targeting, and the development of oncolytic virus therapies.

## 2. Specificity and Tools of the Gene Therapy Applied to Pancreatic Cancer

### 2.1. General Strategy and Experimental Models

There are several specificities of pancreatic cancer gene therapy. First, it requires not only efficient vectors but also capable of transferring large amounts of genes that do not necessarily have prolonged expression. It may also be noted that the inflammatory reactions associated with the use of certain vectors are of lesser importance compared to other indications insofar as the drug genes are not intended to be administered on a regular and continuous basis. The anatomic localization of pancreas is of importance due to a profoundly localized organ with a close relationship with abundant vascularization and surrounding organs. For a clinical point of view, imaging techniques have also of importance to diagnose and perform staging PDAC. Among them not only computed tomodensitometry but especially endoscopic ultrasound (EUS) have a crucial role. EUS combined endoscopy and high-resolution echography by means of a high frequency ultrasonic device placed at the tip of this endoscope. Fine needle aspiration of pancreatic lesions can be performed under ultrasound imaging and nowedays EUS is the major technique to obtain a cytopathological confirmation of PDAC. By means of the same needle intratumor injection of therapeutic compounds can be easily and safely done such gene therapy products [12].

It is well known that pancreatic cancer cells are aggressive with a high rate of proliferation and a powerful local invasive and metastatic properties (peritoneum, lymph nodes, liver, etc.). An over-expression of growth factors and/or their receptors (EGF, nerve growth factor, gastrin) as well as of pro-angiogenic factors (VEGF, FGF, Platelet Derived Growth Factor), and increased invasive factors (metalloproteinases, tissue plasminogen activators) participate to these proliferative and aggressive properties. In addition, this aggressiveness occurs whatever the size of the tumor with a special role of the tumor microenvironment. Indeed, this microenvironment plays also a key role in the invasive and metastatic process of pancreatic carcinoma, with a strong relationship between cancerous cells and pancreatic stellate cells, as well as the extracellular matrix. It strongly participates in tumor fibrosis, hypoxia, and hypovascularization, resulting in inaccessibility of drugs [13,14]. The genetic background of PDAC is well known and *INK4a/ARF* (*p16*), *TP53*, *DPC4/Smad4* tumor suppressor pathways are genetically inactivated in the majority of pancreatic carcinomas (associated with losses of heterozygosity of, respectively, 9p21, 17p, and 18q), whereas oncogenic *Kras* is activated [15,16,17]. At a late stage of tumor development, there is an increase in telomerase activity. However, the activating point-mutation of the *Kras* oncogene on codon 12 (Exon 2) remains the major event (70–95% of PDAC cases: 71% of pancreatic cancer specimens in the COSMICS database harbour Kras mutations) [17,18,19].

Most of models used for pre-clinical approaches of gene therapy are well-established human pancreatic cancer cell lines that can be implanted in athymic mice heterotopically or orthotopically. One limitation of these models is the lack of immunity despite a stromal reaction arising from mouse itself. Other in vivo models are transgenic ones including the mouse model displaying a G12D *Kras* mutation (*Kras* LSL-G12D/+ i.e., KPC model) [20]. In addition, crossed models are also available with animals bearing a knockout of TP53 or other tumor suppressor genes [21,22]. However, transgenic models take often time to obtain primary PDAC and its environment and the immunity remains a mouse context [20,21,22]. Finally, humanized models such as PDX (patient-derived xenograft) could be theoretically applied but require preliminary characterization in term of differentiation, somatic mutations, chemosensitivity or resistance. In our team, we have largely used an immune model with phenotypic and genotypic properties close related with the human model, i.e., orthotopic allotransplantation or portal injection of pancreatic cancer cells in hamster. It is a rapid and reproductive model but the molecular tools for studying hamster are quite poor [23,24,25].

On the other hand, we demonstrated many years ago that human pancreatic cancer cells are difficult to transfect using various synthetic vectors and also adenovirus [23,24]. The use of viral vectors offers generally a better yield of DNA transfection and expression but they cannot be used optimally in all models as specific receptors are sometimes required and human viruses do not necessarily work in rodent cells and organs. Last, two points are peculiarly important when one can consider a gene therapy strategy for PDAC: (i) the evolution of 80% of tumor is rapid with a median survival reaching 8 to 11 months [1,3,4,5] requiring to take into account the fact that chemotherapy should be often included in the therapeutic strategy (before as a first line or in combination with gene therapy) and that the time to set up any second (and hopefully) lines treatment is very short with an otherwise aggressive and often metastatic disease; (ii) the choice of optimal gene, vector and route (frequency) of future administration at the clinical stage should be minded and investigated as soon as possible and thus integrated in the different stage of the clinical development “proof of concept, pre-clinical experiments (up-scaling of gene therapy production, biodistribution, toxicology, etc.) and phase 1.

### 2.2. Methods of Gene Delivery

The three main methods are ex vivo, in vivo and in situ gene therapy. Ex vivo gene therapy involves sampling cells from the patient, genetically modifying them with a vector carrying the therapeutic gene and then reintroducing them in the same patient [26]. This method applies very well to blood stem cells as it is clinically used in the gene therapy protocols for combined immunodeficiency syndroms or hemoglobinopathy [27,28]. This strategy requires an ex vivo culture as currently practice in the context of cell therapy with adult stem cell sampling and more commonly in heterologous bone marrow transplantation. This took times and several controls within at least four to eight weeks procedures. In the context of a tumor like PDAC with a rapid growth and invasive process the ex vivo strategy is not adapted and a gene therapy product “ready to use” is theoretically better. The second type of gene therapy is the in vivo approach. It involves injecting the vector carrying the therapeutic gene directly into the bloodstream. In this case, the injection is easy (it is an intravenous or intra-arterial injection) but several additional barriers are raised like the circulating blood and the vessels. Indeed, in the circulating blood, several actors will try to eliminate the foreign DNA and its vector such as macrophages and endonucleases. Finally, if the transgene must reach an organ, it will have to cross the barrier of capillaries and Endothelial cells. The third type of gene therapy is in situ, delivering the therapeutic gene directly within the target tissue (or tumor). This technique is tested in particular in cases of cystic fibrosis (transfer of vectors in the trachea and bronchial network with aerosols), myopathy (for example injection into the muscle of a vector carrying the dystrophin gene in the myopathy of Duchenne) and cancers in order to inject into the tumor a vector carrying a therapeutic gene. This approach requires two things: a good targeting of the organ and an efficient vectorisation system. In terms of targeting, these are mainly “physical” methods that can be carried out by the hand of doctors or surgeons by means of a simple needle or catheters. In case of PDAC, therapeutic gene injection is guided by surgeon during laparotomy, by the radiologist under ultrasound or CT-scan and last (and better) by a gastroenterologist under EUS which is the most precise technique to target primary PDAC whatever its localization within the pancreatic gland.

This route seems optimal for PDAC gene therapy but require also a specific and elective tropism of the gene therapy product for PDAC cells. This suppose specific promoter as a “molecular targeting” or a specific viral tropism. Molecular targeting is theoretically possible, so far we use specific promoters of PDAC cells. Unfortunately, there is no highly specific promoter even if mesothelin and MUC-1 appear the best candidates to obtain an elective transgene expression in PDAC cells. Finally, the choice of in situ gene therapy appears the best one for PDAC.

### 2.3. Vectors

Besides naked DNA, that do not offer a significant advantage for cancer gene therapy, there are synthetic vectors which have the advantage of being easy to produce by the chemical industry. They are relatively innocuous (although some cellular toxicity in vitro is often found) and interesting properties for gene therapy. Indeed, all the nucleic acids can be complexed (such as plasmid DNA, siRNAs, shRNAs and even some viral vectors). There are two main types of synthetic vectors: lipid vectors and polycationic vectors [29].

The pure lipid vectors have the power of enveloping the DNA and facilitating its passage through the lipid bilayer constituted by the cell membrane. These vectors did not appeared efficacious to produce significant gene transfer into human PDAC cells. They rapidly evolved towards mixed vectors associating a lipid part and a cationic part. The principle of the cationic part is very simple: it is a succession of positively charged molecules which envelop and complex with DNA which is negatively charged. The vector envelops the DNA in order to facilitate the membrane passage by the lipid part and to be protected during its “travel” in the cytoplasm towards the nucleus thanks to the cationic part of the vector. Finally, if mixed vectors displayed higher performances than pure lipid vectors to induce a significant DNA transfection in PDAC cells. A second type of synthetic vector appeared more interesting. Indeed, polycations or cationic polymers are vectors derived from polylysine and polyethylenimine. The polylysine vector is therefore a basic amino acid polymer, lysine, which has the power to condense DNA and protect it from digestion by intracellular lysosomes. Certain chemical groups such as polyethylene glycol (PEG) have been added to improve stability in the blood and the internal environment, or even to decrease cell toxicity.

Among the polycationic vectors, we also find that of the polyethylenimine (PEI) type which is a pure polycation, linear in its structure and which facilitates the cellular and nuclear penetration of the DNA by conferring it a good resistance to lysosomal digestion. Several vectors are derived from the PEI and have parts added by chemists such as PEG, cholesterol, mannose and dextrose and many other chemical compounds [30,31,32,33,34,35]. A number of these DNA-complexed vectors have been tested in patients and have not had any major adverse effects at any site of administration (lung cancer, subcutaneous vaccination, ovarian cancer, lymphocytes or bladder cancer). Clinical studies have been conducted in PDAC patients (including our experience) using PEI and with safety and possible efficacy pending results of ongoing phase 2 trials. Finally, it should be noted that the DNA-polycation complexes take a final structure and especially a size close to the manometer. As a result, they are also called “nanocomplexes” or “DNA nanoparticles”.

Besides synthetic vectors, viral vectors have been developed for their natural property to infect human cells [29,36,37] (Table 1). Adenoviruses are responsible in children and adults of head and neck, pulmonary and digestive infections. They infect many cells and, as a result, vectors derived from adenoviruses have long been used in preclinical and clinical gene therapy strategies for PDAC. They also induce transient expression and of provoke strong inflammatory and immune reactions. However, several clinical trials have been conducted with adenoviral vectors, without major incident. In order to overcome some problems of toxicity, third generation adenoviral vectors lacking viral sequence have been developed (so called “gutless” or “helper dependent”) [36,37,38]. Adeno-Associated Viruses (AAV) are non-pathogenic, naturally defective viruses that are widespread in humans. AAVs are derived from viruses of the parvovirus family. They are used as single-stranded DNA vectors, are very small in size, unwrapped and transduce quiescent cells and dividing cells. They only rarely and randomly integrate and therefore present a low mutagenic risk. They are termed “associated adenoviral vectors” because the parental viral strain, the adeno-associated virus, depends, for replication, on the presence of adenovirus, herpes simplex virus or papillomavirus. They can effectively transduce cells from the brain, liver and some blood cells but have been rarely tested in human PDAC cells. Retroviral (most derived from MuLV for Murine Leukemia Virus) vectors and lentiviral (derived from HIV-1 for Human Immunodeficiency Virus Type 1) belong to retroviridae family. These are small enveloped RNA vectors which have the advantage of efficiently transducing a lot of cells of interest, being not very immunogenic (and without any pre-existing immunity because they are very rare in nature) and which are easy to produce. Nevertheless, the weak point of this type of vectors is their integration in open areas of the chromatin of the nucleus of the target cell, which presents a risk of mutagenic insertion (they can be considered as onco-retroviruses). They are mainly used in ex vivo strategies but their ability of the transduction of proliferating cells such cancer cells authorize also an in situ administration [27,28,37,38]. A new generation retroviral and lentiviral vectors have been modified to avoid the transactivation of oncogenes when they are integrated into the host cells: these are the so-called SIN (self-activating vectors, avoiding transactivation of oncogene such as LMO oncogene). Another type of modified lentivirus has been also successfully tested by our team in PDAC models using a integrase-defective lentiviral vectors [39].

Some researchers have studied herpes viruses as gene therapy tools, such as HSV-1. It is the opposite of the AAV virus from the point of view of its advantages and disadvantages. Indeed, HSV-1 virus can carry large amounts of DNA but it is highly immunogenic (and human HSV-1 antibodies frequently). On the other hand, this virus has a strong tropism for the nerve cells in contrast to most other viral vectors. However, its genome is complex and the passage from a highly pathogenic virus to a clinically effective harmless vector is far from simple. Other viruses have been used for in vitro and in vivo gene transfer and are derived from other pathogenic viruses such as measles, vaccinia, pox or Sendai virus. Their application in gene therapy remains marginal but a development is currently observed in strategies of vaccination (pox virus) or in vivo administration (vaccinia virus). Main viral vectors and their characteristics are detailed in Table 1. In addition some authors add to this list the concept of “biological vectors” taking into account that attenuated strain of bacteria can be used for gene therapy (and vaccination protocols) by introducing therapeutic transgene [38,40]. Finally, it is noteworthy that the field of gene transfer has been considerably opened since the nanotechnologies and nanomaterials have been adapted to DNA transport [41].

The route of administration depends on the type of vectors. Generally adenovirus, AAV and synthetic vectors are used for in situ administration, retroviral and lentiviral vectors are applied for ex-vivo approaches. Oncolytic viruses can be injected by IV route while subcutaneous injections can be performed with plasmidic DNA as well as transfected cells or bacteria. Whatever the system of gene delivery and in case of solid tumors such as PDAC it is difficult to reach all tumors cells as doing chemotherapy. The in situ approach can benefit from a radiological targeting but on cannot really know how many cancer cells are transfected. In the vivo approach gene transfer can be tone down by natural barriers such as blood stream, nucleases, capillaries or tumor stroma. Once again the best way would be a “robust” molecular targeting (promoter driving the gene of interest or molecular motif within the vector itself) specific of pancreatic cancer cells.

## 3. Therapeutic Genes and Pre-Clinical Strategies

The general strategy and tools for gene therapy of cancers will vary depending on the final goal: either to reintroduce a deficient gene (such as transfer of a tumor suppressor gene) or to introduce a missing or under-expressed therapeutic gene (such as a cytokine or a suicide gene) or to inhibit the expression of a gene (such as an oncogene). On the other hand, whatever the strategy of administration (in vivo or in situ), one should not expect to reach a large part of the tumor in terms of gene transfer and expression. Indeed, many successive obstacles are to be overcome such as access to tumor cells, intracellular and intranuclear penetration, expression of mature mRNA and protein. In our experiment by applying a plasmid vector complexed to PEI or a recombinant adenovirus, we obtained a maximum of only 15% of the tumor cells of the primary tumor after administration in situ [23,24,25]. This percentage implies to obtain a bystander effect in order to hopefully induce a significant antitumor effect. The bystander effect realizes a diffusion of the therapeutic effect to the neighboring cells following the transfer of the therapeutic gene. This prerequisite must be observed in any proof of concept for gene therapy program of epithelial cancers. This bystander effect has been described initially after in vitro and in vivo transfer of suicide genes. It generally involves the diffusion of pro-apoptotic signals, activated prodrugs and even anti-angiogenic paracrine signals. The various pre-clinical approaches of gene therapy for the PDAC are detailed in Table 2 and developed in the sub-chapters below.

### 3.1. Tumor Suppressor Genes

This strategy aims to obtain the transduction of an antioncogene, the expression of which has been lost during carcinogenesis. The most well-known tumor suppressor gene is that encoding TP53, a protein that controls the entry of apoptosis cells that are mutated in 50% of human tumors. The direct in vitro inhibitory effect of the restoration of TP53 expression on tumor growth is complemented by an in vivo inhibitory action on neoangiogenesis. This *TP53* gene transfer strategy has been applied to the treatment of tumors of the liver, stomach, colon, lung, ovary and head and neck cancers. The results obtained are poor and often reflect a poor efficiency of gene transfer in vivo, but also differences in the sensitivity of cell types or even the inconstant mutation of TP53 in tumors in general. However, it should be noted that the first gene therapy medication authorized for the treatment of cancers involves the transfer of the *p53* gene (see below in chapter on oncoviruses). Beside *TP53*, others tumor suppressor genes has been tested in vitro and or in vivo in PDAC models such as *p21WAF1*, *p16INK4A*, or *DPC* [38,42,43,44,45,46,47,48].

### 3.2. Antiangiogenic and Proapototic Genes

Always in vitro and in vivo models, several strategies has been tested by transfer of gene encoding for anti-angiogenic molecules such as endostatin, angiostatin, thrombospondin-1, vasostatin, soluble fibroblast growth-factor receptors as well as soluble vascular endothelial growth factor receptor [38,49,50,51,52,53,54,55]. As a proof of concept of a future gene therapy protocol for PDAC (see chapter clinical trials) we demonstrated that by re-introducing the somatostatin receptor subtype 2 (*SSTR2*) gene (the expression of which is lost in PDAC), we obtained in a mouse and hamster models a local and distant antitumor bystander effect. This bystander effect is mediated by apoptosis and antiangiogenic effect through a negative autocrine loop induced by gene transfer with secretion of the natural ligand somatostatin [23,24].

### 3.3. Suicide Genes

An important issue is that a gene transfer of a suicide gene with a strong neighboring antitumor effect (so called the “bystander effect”) may compensate for the weakness of gene expression within the tumor. The most classic example of this suicide gene strategy is that of the herpes virus thymidine kinase gene (the *HSV-TK* gene). This gene encodes the thymidine kinase enzyme which is able to metabolize ganciclovir, an antiviral drug that is normally devoid of antitumor effect, into a toxic compound that interferes with DNA replication and results in cell apoptosis. This system provides conditional toxicity based on the presence of the sensitizing gene and its substrate. In other words, the gene is theoretically harmless to the cells in which it will be expressed but becomes highly toxic when its substrate, ganciclovir, is added. The latter is also harmless in its initial form (before administration) but will become toxic in the cell after its phosphorylation by thymidine kinase. Using this enzyme for therapeutic purposes, it was quickly apparent in vitro that its expression in only 10% of the cell population was sufficient to cause the death of all cells in culture after treatment with ganciclovir. Several studies have demonstrated that this remote toxicity was due to the phosphorylated release of ganciclovir from cells expressing the HSV-TK enzyme to those that do not express it, via intercellular communications. Pro-apoptotic messages and enzymes would also be diffused from cells expressing the *HSV-TK* gene. In vivo, this system also induces a strong immune response directed against tumor cells. Indeed, it is suspected that the fact of destroying the tumor cells releases tumor antigens that will lead to a response of the body against other tumor cells. This is called a distant bystander effect [56,57,58]. The other examples of suicide/prodrug gene systems that has been also successfully tested in PDAC models are as follow: the *cytosine deaminase* gene (also coupled to the uracil phosphoribosyltransferase gene) that transforms 5-fluorocytosine (with antifungal properties) into 5-fluorouracil [38,59,60]; the *nitroreductase* gene transforms CB1954 (for [5-(aziridin-1-yl)-2,4-dinitrobenzamide]) into a toxic compound, the 4-hydroxylamine [61,62]; the *cytochrome P450* gene that transforms ifosfamide to acrolein (nitrogen mustard);. At a clinical point of view, the cytochrome P450/isofosfamide system has been developed crossing in vitro and in vivo proof of concept in order to subsequently conduct a phase 1 trial in PDAC patients (see also chapter clinical trials) [63,64].

### 3.4. Small Non-Coding mRNA, Interferent mRNA and Antisense Therapy

Besides the conventional gene transfer using vectorized DNA (synthetic or viral vector), there is another strategy, acting directly on the RNA and thus shunting the DNA and its transcription process. Small non-coding RNAs have been discovered since the late 1990s and two-classes of small RNA, the microRNAs (miRNAs) and interfering RNAs such as siRNAs (for “Small Interfering RNA”) and shRNAs (for “Short Hairpin RNA”). These non-coding RNAs have many functions, in particular the post-transcriptional inhibition of gene expression.

MicroRNAs (or miRNAs) are single-stranded RNAs of 21–24 ribonucleotides specific. They are natural molecules endowed with a post-transcriptional regulatory power and therefore capable of extinction of the expression of a gene. Indeed, their pairing with a sequence complementary to the target messenger RNA can lead to the inhibition of the translation of the mRNA or its degradation. They were notably involved in the modulation of processes such as apoptosis, cell cycle or differentiation. Loss of their expression (by chromosomal impairment, mutation, polymorphism, epigenetic modifications) can lead to major dysfunctions, especially during carcinogenesis. The study of the expression on a large scale (called the microRNome) made it possible to determine the role of some microRNA in the development of many cancers as well as the mechanisms that lead to these dysfunctions. These miRNAs so-called oncoMIR can be down regulated through a gene therapy approach. We successfully demonstrated that targeting the oncogenic miRNA-21 (down regulation by an antisense expressed with a lentiviral vector) strongly inhibit pancreatic cancer tumor growth both in vitro and in vivo [65].

An interfering RNA is a single or double-stranded RNA (composed of 21 ribonucleotides) whose interference with a specific mRNA leads to its degradation and to the reduction of its translation into protein. Like micro-RNA, the interfering RNA makes it possible to block translation by making “silent” this or that gene. To this arsenal must be added the small hairpin or shRNAs (shRNAs for short hairpin RNAs), the structure of which is in the form of a stem and a loop, which are also involved in the phenomenon of Interference with RNA. The expression of the shRNAs in cells is carried out mostly by means of plasmids or viral vectors. Finally, using the RNA tool avoids the nuclear compartment of the cell (unlike naked or vectorized DNA) and therefore all transcriptional and post-transcriptional machinery. Some successes in vitro and in vivo have been recorded and several pre-clinical and clinical trials currently apply the strategy of interfering RNA. In the same class of tools that directly address gene expression, there is the antisense strategy. It is indeed possible to synthesize a nucleic acid strand (DNA, RNA or a chemical analogue) intended to bind to the mRNA of the target gene when it is expressed. This has the effect of inactivating the gene (sometimes destroying it). It is also possible to affect the splicing of the pre-RNA, thereby changing the exon content of the mRNA. The synthetic nucleic acid sequence used is called “antisense” because it is complementary to that of the messenger RNA of the gene, which is called the “sense sequence”. It is, therefore, possible to synthesize oligonucleotides to block the expression of a deleterious gene by its expression or overexpression (such as an oncogene, a growth factor, pro-inflammatory molecules, mediators, etc.) [66].

This strategy has been applied to PDAC both at the experimental and clinical levels targeting *Kras* oncogene expression with antisense, siRNA and ribozymes molecular tools. Unfortunately clinical results did not reflect those positively obtained in vitro [19,38,67,68,69,70]. A compound is now evaluated at the clinical stage, the LODER^®^ (Silenseed Ltd., Modi’in, Israel) that is a miniature biodegradable intratumoral implant containing and releasing within 4 months siRNA against *Kras*.

A recent most sophisticated approach, is genome editing that encompasses a set of techniques for manipulating the genome via “rewriting genetic material” that can be applied to plants, animals, fungi and bacteria. Some laboratories also propose to apply it to the human genome and use it as a gene therapy tool. Three new systems have potential in biomedical research and perhaps in personalized medicine. The first two use endonucleases as Zinc-Finger Nucleases (ZFN) and Transcription Activator-Like Effector Nucleases (TALEN). The third system is considered to be a real technological revolution, the system CRISPR-Cas9. Known since 2012, it comes from a system of bacterial defense. Indeed, bacteria integrate into their genes, at the level of the CRISPR (“Clustered Regulatory Interspaced Short Palindromic Repeats”) sequences, foreign DNA fragments, as a “souvenir” of past infections. These latter are transcribed into RNA, which can then guide the enzyme Cas9 (for “CRISPR associated protein 9” which is also an endonuclease) to the foreign DNA when it presents itself in order to eliminate it. On this principle, it is possible to obtain a CRISPR-Cas9 artificial complex specific for a given DNA sequence (generally between 10 and 24 nucleotides) in order to cut and modify it. Of course, this is an extraordinary tool for molecular biology but also for gene therapy: to remove a specific deleterious sequence, to replace it but also to add a sequence of crucial interest, in short, to modify the genome at will [71,72,73]. This strategy is already applied in gene therapy approach of HIV infection, paludism or pulmonary cancer [74]. An in vivo gene transfer of this system is possible in various models of cancer including PDAC [75]. One question remains to resolve, which crucial gene should be targeted to reduce cell proliferation and/or invasion? Besides the specificity of the gene (and a lot of candidates may be chosen in the case of PDAC) some milestones must be passed such as accuracy, efficiency and safety of CRISPR-Cas9 technology applied for a gene therapy strategy.

### 3.5. Immunotherapy and Vaccination

The lack of recognition and elimination of tumor cells by the immune system is strongly implicated in the development and progression of tumors. This is one of the explanations for the aggressiveness of certain cancers. Indeed, PDAC is characterized by the accumulation of a desmoplastic stroma rich in inflammatory cell infiltrates [76]. While immune cells are abundant within the stroma, they mostly belong to immunosuppressive subsets, such as regulatory T cells (Tregs), T-helper (Th)17 cells, tumor-associated macrophages (TAMs) and multiple subsets of immature myeloid cells/myeloid-derived suppressor cells (MDSCs) [77,78,79]. Intratumoral effector T-cells are rare, in contrast to many other solid tumors, and when present express high levels of immune checkpoint receptors such as PD-1, indicating an exhausted status. As observed in many cancers, PDAC is subjected to a microenvironment remodeling to survive. PDAC specifically promotes an immunosuppressive microenvironment and a pro-tumoral inflammation. One can underline the specific role of PDAC stellate cells with a paracrine signaling mediated by cytokines such as GM-CSF (Granulocyte-macrophage colony-stimulating factor) and IL-6 [80]. In addition, cancer cells themselves expressed checkpoint molecules (such as programmed death ligand-1/PDL-1) [81]. A limited number of studies targeting theses immune checkpoint inhibitors (ICI) have been completed in PDAC. Both Cytotoxic T-Lymphocyte Associated Protein 4 (CTLA-4) and Programmed death-ligand 1 (PD-L1) inhibitors were investigated in patients with locally advanced or metastatic PDAC in two clinical trials. Unfortunately, PDAC is not melanoma and the clinical outcomes were disappointing [77,82]. On the whole PDAC microenvironment is comprised of an intricate network of signals between immune cells, stellate cells and PDAC cells resulting in an immunosuppressive environment resistant to single-agent immunotherapy including gene therapy [77,81]. 

There are many strategies of immunotherapy applied to the treatment of cancers. The more classical is the non-specific immunotherapy aiming to administrate cytokines with antitumor effect (interferons, interleukins, etc.). To overcome some side effects provided by systemic administration gene therapy strategies has been developed to induce local interleukin or cytokine either by the tumor cells themselves or via ex-vivo approach (example of human fibroblast transduced with interleukin-12) [38,83,84,85,86,87,88]. Pre-clinical experiments have been conducted in vitro and in vivo aiming to produced interleukins (IL-2, IL-4, IL-6, IL-27, IL-1β, IL-24) and cytokines (GM-CSF, IFNα and β, TNFα). Most of experiments were positive with antitumor effect as proof of concept of future clinical trials.

In the field of specific immunotherapy, there is several routes. The first one is the administration of monoclonal antibodies to receptors or molecules involved in the growth, immunity or vascularization of tumors. The second one is the adoptive immunotherapy by introducing a tumor antigen into the lymphocytes. It consists in stimulating the patient's immune system against his own tumor so as to facilitate the recognition of cancer cells and their elimination. The antigen (peptide or gene transfer) is introduced in the T lymphocytes or antigen-presenting cells (so called “dendritic cells”) of patients. The gene therapy approach consists in introducing a gene encoding a protein involved in tumor cell recognition, in their destruction (tumor antigens, cytokines, tumor suppressor gene) and to reinsert them into the organism of the patients. Antigens commonly expressed for PDAC are MUC-1 (Mucin-1) and mesothelin. Antigen-pulsed dendritic cells vaccine experiments have been conducted in PDAC models. In term of adoptive active immunotherapy of cancers, a new approach has been developed. It is based on the principle of the Chimeric Antigen Receptors. Autologous lymphocytes of the CD8+ T cell type are removed from the patient and are modified to recognize and kill the tumor cells carrying tumor antigen. By means of a lentiviral (or retroviral) vector, a monoclonal antibody fragment (in fact several fragments, hence the term “chimeric”) is transduced into the patient's lymphocytes and then reinjected. Some initial tests for malignant hemopathies such as acute B cell lymphocytic leukemia, lymphocytic B cell lymphoma and chronic lymphocytic leukemia are promising and first trials are in progress for PDAC [89,90,91,92,93].

The last approach of immunotherapy is vaccination by systemic administration (i.e., intravenous infusion), subcutaneous or local administration of tumor antigens in the form of a peptide, a protein, a tumor cell lysate, a DNA plasmid or a recombinant viral vector. Several programs have been initiated and reached the clinical application during early phases. Beside antigen-pulses dendritic cells, various strategies are developed such as: DNA vaccine with the VEGF-receptor DNA, peptide vaccination (Gastrine17-DT vaccination, mutated peptides of Kras), CRS-207 (attenuated form of Listeria monocytogenes that has been genetically-modified to express mesothelin), TELOVAC (vaccination trial using a 16-amino acid telomerase peptide - GV1001) and GVAX (mix of allogenic human pancreatic cancer cells expressing and secretin GM-CSF) [38,94,95,96,97,98,99,100]. Most of the strategies of vaccination are associated with GM-CSF administration and/or chemotherapy. 

## 4. Oncolytic Virotheray

Development of PDAC is associated with a series of changes that provide a selective growth advantage to the cancer cells. However, these very changes (among them, lack of interferon response, elevated metabolic activity, and disengagement of cell cycle control) render cancer cells highly sensitive to viral infection. Consequently, selectively replicating viruses (oncolytic viruses) represent a promising new therapeutic strategy. These viruses can be naturally “cancer selective” for their replication or may require genetic modifications. A recent clinical-trial-watch report has summarized the use of oncolytic virus for cancer therapy [101]. Oncolytic viruses induce their anticancer activity through several mechanisms such as a preferential replication in dividing tumor cells until apoptosis and cell death, tumor cell specificity, the targeting of dysfunction pathway (such as *TP53*), the induction of specific and non-specific anti-tumor immunity and last suppression of cancer-stem cells [102]. Oncolytic viruses are natural pathogens that have been selected or specifically designed to infect and destroy cancer cells. These oncovirus are under development in academic and pharmaceutical laboratories around the world for more than twenty years. They can be engineered to produce cytokines, antigens or suicide genes.

The first oncovirus that has been approved for clinical indication is an adenovirus, Oncorine H101 (developed by Shangai Sunway biotech) that is engineered and modified selectively to replicate in and kill tumor cells that harbor p53 mutations. It is close related to the adenovirus Onyx-15. The clinical indication is head and neck cancers but Onyx-15 has been evaluated in PDAC. The second one is an herpes virus (HSV-1), OncoVex GM-CSF (T-VEC, Talimogen laherparepvec, Imlygic^®^ developed by Amgen, Thousand Oaks, CA, USA) was approved for the treatment for advanced melanoma by FDA (US Food and Drug Administration) and EMA (European Medicine Agency). Others oncoviruses reached the stage of clinical evaluation such as JX-594 (developed by Jenerex, a vaccinia virus expressing GM-CSF) tested in hepatocellular carcinoma, Reolysin^®^ (Oncolytics Biothech Inc., Calgary, Canada) (a reovirus for “respiratory enteric orphan virus”) tested in brain tumors and PDAC, Toca 511 (a retroviral replicating vector encoding a modified yeast cytosine desaminase in combination with 5-fluorocytosine developed by Tocagen) tested brain tumors but also in PDAC, the TG4023 (vaccinia virus encoding for expressing cytosine deaminase and uracil phosphoribosyltransferase in combination with 5-fluorocytosine developed by Trangene) tested in hepatocellular carcinoma and hepatic metastasis [103].

In the case of PDAC, several viruses have been explored for their ability to replicate and to inhibit tumor growth in experimental model [37,104,105]. Preclinical studies have demonstrated that conditionally replicative adenoviruses can be exploited for PDAC therapy, when combined with gemcitabine [106]. Pioneering studies demonstrated that intratumorally injected Onyx-15 combined with gemcitabine was well tolerated in a phase I/II clinical trial for PDAC patients [107]. Current refinements in conditionally replicative adenoviruses as a result of a German–French collaboration comprise modifications of the hexon to improve viral replication in both tumor and stromal cells [108], as PDAC is characterized by a dismal stromal reaction that impedes the intratumoral dissemination of therapeutic drugs. Interestingly, growing body of evidences indicate that PDAC tumor microenvironment is not a watertight barrier, but rather facilitates oncolytic viruses replication and therapeutic activity [109].

We recently demonstrated that engineered herpes simplex type 1 viruses (HSV-1) are efficient in blocking experimental tumor growth, used alone or in combination with gemcitabine [110]. HSV-1-based viruses infect various tumor cell types, do not integrate into the genome of infected cells, are safe in patients, and there are several anti-HSV-1-specific drugs available in case of adverse events. In addition, cell killing of entire cancer cell populations can be achieved rapidly with a relatively low dose of virus. The most widely used strategy to restrict HSV-1 replication to cancer cells is to delete genes that are important for viral replication as well as the evasion of innate immune responses. First-generation oncolytic HSV-1 viruses, such as Oncovex and HF10, which have reached clinical applications, were ablated for the γ34.5 viral protein and resulted in reduced therapeutic efficacy [105]. In our work performed by the HSV-1-based virus, Myb34.5, in which the expression of the γ34.5 viral protein is controlled by a tumor-specific B-Myb promoter, efficiently replicates and kills human PDAC cells. When injected in orthotopic human tumors developed in nude mice, Myb34.5 successfully replicates, strongly inhibits tumor progression and blocks metastatic dissemination. The Myb34.5 antitumor effect is potentiated by gemcitabine administration, both in vitro and in vivo [110].

The oncolytic parvovirus, H-1 (H-1PV), belongs to the Parvoviridae family and is composed of a small, non-enveloped, icosahedral capsid containing a linear, single-stranded DNA around 5 kb in size. The mechanism underlying the natural selective replication as well as the specific toxicity of the virus for rat and human cancer cells is complex and multifactorial. If H-1PV binds and infects equivalently normal and transformed permissive cells, it is thought that the molecular abnormalities encountered in cancer cells favor nuclear envelope breakdown, viral gene expression, viral DNA amplification, capsid assembly, virion maturation and cytopathic effect. Variable levels of constitutive activation of the specific metabolic/signalling pathways leading to these cellular events may explain the difference in potency of H-1PV, as permissive, semi permissive and a few resistant cancer-cell types have been described [111].

H-1PV efficacy and safety was tested in a rat orthotopic model of PDAC, involving either rat cells in a syngeneic rat or human carcinoma cells in an immuno-incompetent model [112,113,114]. Other experiments led to similar conclusions on human PDAC tumors that were implanted immuno-incompetent mice, that are non-permissive to H-1PV. The efficacy of H-1PV was also tested in combination therapies, in the context of PDAC. Considering that H-1PV induces cancer-cell killing using a different mechanism (cathepsin dependent), oncolytic H-1PV could potentially kill cells resistant to gemcitabine. Considering the clinical application of H-1PV in patients, a French study, in which the skin metastases of twelve patients with melanoma, breast adenocarcinoma, lung large cell carcinoma, pancreatic carcinoma, or kidney leiomyosarcoma were injected with escalading doses of H-1PV, reported only mild signs of toxicity. The presence of viral DNA and proteins was detected in the injected tumor lesions, as well as at distant tumor sites, suggesting a systemic spread of the virus. In light of the lack of significant toxicity encountered in this early clinical trial, an oncolytic H-1PV trial (ParvOryx01) proposing escalating doses of H-1PV was initiated in patients with malignant brain tumors [113], following injection into the primary site, or intravenous administration, and was found to be safe. Altogether, the clinical trials reported so far involving administration of H-1PV in cancer patients have demonstrated that this virus is safe and devoid of clinically relevant side effects. In addition, the ParvOryx01 trial, which has just been completed and is under current clinical assessment, provided intriguing evidence of viral intracellular replication, induction of viral and cellular cytotoxic effectors, and activation of specific T-cell responses [114], which could be translated in the future to the therapy of pancreatic cancer. A new therapeutic concept is emerging by combining viral oncolysis with immunotherapy. In fact, at the same time, virus mediated tumor lysis leads to the liberation of tumor associated antigens and/or mutant proteins that have arisen during tumor evolution [115]. Imlygic^®^ has provided the first convincing human data supporting the idea that direct tumor lysis by a replicating virus can locally stimulate sufficient anti-tumor immune responses to provide systemic, long-lasting cancer killing immune responses in advanced cancer patients [116]. This oncolytic virus was found to increase T cell infiltration into tumors and generate a systemic immune response against tumor associated antigens [117]. Thus, combining oncolytic virus with immune checkpoint inhibitors to override immune tolerance has emerged as one of the most promising anticancer modality to date. It is tempting to speculate that heating up immunologically cold pancreatic tumors with oncolytic virus will overcome resistance to ICI treatment for best patient benefit. 

Additionally, data from preclinical models and patients suggests that the natural induction of anti-tumor immunity is “hit and miss” with a pure oncolytic approach. Rather than hoping for an immune response to be induced with this strategy, oncolytic virus offers the opportunity to encode foreign transgenes [118] and to create an improved oncolytic virus vaccine for PDAC. In this strategy, a “supercharged” immune response is directed against a specific antigen expressed on the patient’s cells, to create the perfect immunological storm within the tumor leading to profound therapeutic effect both on primary site and metastatic deposits. To explore this hypothesis, oncolytic virus could be generated to express diverse PDAC antigen (mesothelin, MUC-1, etc) that were recently found to elicit a potent antitumoral immune response when combined with GVAX [119] or used in chimeric antigen receptor-T cells strategies [115], respectively. Supercharged oncolytic virus could be assessed for tumor destruction and immune response against the tumors, including in combination with immune checkpoint inhibitors. 

## 5. Clinical Trials

**Figure 1:**
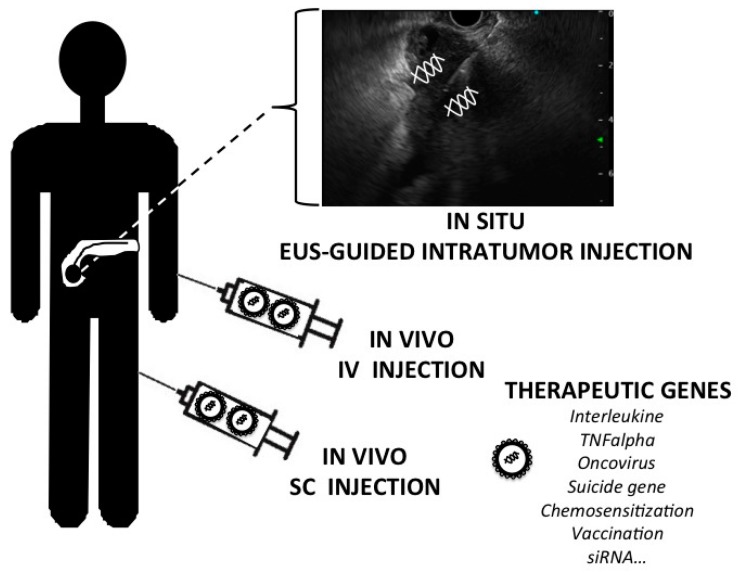
Clinical approaches of gene therapy for pancreatic cancer: type of gene therapy and routes of administration, main therapeutic genes. (EUS: endoscopic ultrasound; IV: intravenous; SC: subcutaneous).

Main clinical trials published and for which we have sufficient data are detailed in Table 3. Main indication is PDAC at an advanced stage (locally advanced and/or metastatic) with phases 1 and 2 trials and one phase 3 [101,120,121,122,123,124,125,126,127,128,129,130,131,132,133,134,135,136,137,138,139,140,141,142,143,144,145,146]. Three types of strategies have been conducted: in situ gene therapy product injection (by means of EUS), vaccination (intradermal or subcutaneous route) and oncolytic virus administration. Gene transfer has been performed either with adenovirus or with synthethic vectors. All treatments have been well tolerated (i.e. no serious adverse event). As observed for pre-clinical studies the transgene provided active immunotherapy with interleukin production but also after suicide genes expression. Oncoviruses were adenovirus Onyx-15, adenovirus H101, herpes virus plus GM-CSF (Imlygic^®^ Amgen, Thousand Oaks, CA, USA) and reovirus Reolysin^®^ Oncolytics Biothech Inc., Calgary, AB, Canada). In most of the programs, patients frequently received chemotherapy or radio-chemotherapy. Gene therapy in these cases can be considered as an “add-on strategy”. All these strategies are schematized in Figure 1. We also developed a gene therapy program, the Thergap Trials, which are based on chemosensitization to gemcitabine. In collaboration with InvivoGen Company, a gene therapy product GMP was produced and tested during pre-clinical phases. We thus devised a novel approach based on intratumoral therapeutic gene transfer to inhibit PDAC tumor growth. We identified *SSTR2* and *DCK::UMK* as complementary therapeutic genes to sensitize cancer cells to chemotherapy, to induce tumor regression and to block metastatic dissemination in experimental models [23,24,25]. We previously demonstrated that in situ gene transfer of *SSTR2* (somatostatin receptor subtype 2 gene) induced a significant antitumor effect (with a bystander effect) together with antiangiogenic properties and chemosensitization to gemcitabine. The *DCK::UMK* (deoxycitine kinase::uridyl monophosphate kinase) fusion gene, by phosphorylating twice intracellular gemcitabine, also induced a significant tumor regression and bystander effect in our relevant models of PDAC. We thus generated a clinical-grade gene therapy product, namely CYL-02, encoding for the above-mentioned genes delivered by non-viral vectors, that was first validated in experimental models. We next administrated CYL-02 to patients with advanced PDAC and demonstrate that gene therapy is feasible and well tolerated without serious adverse events. In selected patients, tumor volume and cancer biomarker levels decreased, metastatic spread was blocked while median overall and progression-free survivals were significantly improved when compared to historical series of patients. We identified circulating Alpha-2-macroglobulin as predictive of treatment response [144]. These promising results warrant further evaluation in selected PDAC patients during an ongoing phase 2 gene therapy trials (Thergap-2 trial, supported in part by the InvivoGen Company (Toulouse, France)—Table 4).

We can conclude from all these trials that the first results are encouraging for most of phase 1 but remain “fragile” in phase 2, in which the difference between standard treatment and gene therapy did not reach high significance. However, that requires trials with larger groups and selected patients such as first line treatment. In Table 4 the main protocol inserted by investigators in the clinical trial web is detailed, recruiting or not, but not published. Besides classical approaches (in situ gene transfer, vaccination and oncoviral therapy) new concepts of adoptive immunotherapy through CAR-T technology reached the clinical stage.

## 6. Conclusions

We have come a long way since the first gene transfer tests in vitro and in vivo. We are now at the stage of clinical trials for which a long and difficult process has to be overcome as with drug development: proof of concept, preclinical safety tests, early phases and phase 2. In addition these are unconventional and genetically modified medication adding a degree of complexity. The severity of the disease requires urgently therapeutic innovations in combination with conventional chemotherapy, the contribution of which is modest. From these therapeutic combinations will surely come the solution. Gene therapy also opens up the field of oncovirotherapy and adoptive immunotherapy that diversifies and strengthens this approach. Open question: Will the future give us reason?

## Figures and Tables

**Table 1:** Main viral vector and their characteristics used in pre-clinical and clinical strategies of gene therapy for pancreatic cancer.

Virus	Insertion Capacity	Target Cells	Delivery	Transgene Expression	Level of Expression	Pre-existant Immunity	Bio-Safety
AdV	35 kb	Dividing or non dividing	Ex vivo or In situ	Transient	High	Yes	Immunogenic, inflammation No integration
AAV	4.8 kb	Dividing or non dividing	Ex vivo or In situ	Stable	Moderate	Yes	Mutational integration
Retrov	8 kb	Dividing	Ex vivo or In situ	Stable	Moderate	No	Mutational integration
LentiV	10 kb	Dividing or non dividing	Ex vivo or In situ	Stable	High	No	Mutational integration, recombination with WT HIV
HSV	30 kb	Dividing or non dividing	Ex vivo or In situ	Transient	High	Yes	Mutational integration, neurotoxicity
Pox	25 kb	Dividing or non dividing	In vivo or In situ	Stable	High	No	Immunogenic, Adjuvant to vaccination
SV40	5 kb	Dividing or non dividing	Ex vivo or In situ	Stable	Moderate	No	Mutational integration

AdV: adenovirus derived vector; AAV: Adeno-associated derived vector; RetroV: retroviral derived vector; LentiV: lentiviral derived vector; HSV: herpes simplex viros derived vector; Pox: Pox and vaccinia virus derived vector; SV40: simian virus 40 (papova virus). AdV, HSV and Pox are double stranded DNA virus; AAV is a single stranded DNA virus; RetroV and LentiV are single stranded RNA virus. WT: wild type.

**Table 2:** Main experiments and strategies tested for gene therapy of pancreatic cancer.

Strategy	Detailed Genes
Gene transfer	Tumor suppressor genes (*P16*, *p21*, pRb, *p53*, *Smad4/DPC4…*) Ani-angiogenic genes (endostatin, thrombospondin-1, angiostatin, Matrix metalloproteinase inhibitors, somatostatin receptor sst2 subtype, arrestin…) Apoptosis related genes (*TRAIL*, *TNF*) Suicide genes
Gene invalidation	antisens therapy (*Kras*, *HER-2/ErbB-2*, *MDR-1*) MicroRNA (MIR-21)
Active immunotherapy	Interleukin expression Cytokine expression
Vaccination	Pulsed dendritic cells DNA, peptide, engineered cells and bacteria
Adoptive immunotherapy	CAR-T cells (targeting mesothelin or MUC-1)

**Table 3:** Main published clinical trials of gene therapy for pancreatic cancer.

Author, Year [Reference]	Phase, (Patient Number-StAge III–IV)	Route	Vector/Strategy	Results
Gilly et al., 1999 [120]	I/II (7)	IT, during surgery	AdV/Interleukin 2	Well tolerated, 1 tumor regression
Mulvihill et al., 2001 [121]	I (3)	IT (CT, surgery)	AdV/ONYX-015	No objective response
Löhr et al., 2001 Salmons et al.,2003 [122,123]	I/II (14)	angiography	Lipofectamine/Cyto. P450 (*)	Well tolerated, 2 PR, 12 SD, OS survival: 10 months
Pecher et al., 2002 [124]	I/II (10)	SC	Cationic liposome/dendritic cells transfected with MUC1 cDNA	No side effects, 9 PD, 1 SD
Hecht et al., 2003 [107]	I/II (21)	IT, EUS-guided	AdV/ONYX-015 + gemcitabine	2 duodenal perforations, 4 PR, 6 SD, 11 PD
Gordon et al., 2003 [125]	I (3)	Intravenous	Rv/Rexin-G	Well tolerated 2 SD (4, 5 months) 1 PD (21 months)
Sangro et al., 2004 [126]	I (7)	IT, EUS-guided	AdV/Interleukin 12	Well tolerated, SD
Senzer et al., 2004 [127]	I (30)	IT EUS-and CT-guided	TNFerade	22% fever, 19% chills 5 complete responses and 16 PR
Mazzolini et al., 2005 [128]	I (11)	IT, EUS-guided	Autologous dendritic cells transfected with an Adv encoding interleukin-12 gene	Well tolerated, 1 PR, 2 SD, 8 PD
Kaufman et al., 2007 [129]	1 (10)	ID	Vaccination with Vaccinia and Pox virus expressing CEA MUC-1) and co-stimulatory molecules	10/10 Antibody responses against vaccinia virus 6, 3 months of OS
Galanis et al., 2008 [130]	I (12)	IV	Rv/Rexin-G	Well tolerated, no evidence of anti-tumor activity
Laheru et al., 2008 [131]	I (50)	ID	Plasmid/GVAX Arm A: GVAX alone Arm B: GVAX with cyclophosphamide	Well tolerated, Median survival : A: 2.3 months B: 4.3 months
Chawla et al., 2010 [132]	I/II (9)	IV	Rv/Rexin-G at two dosages	Well tolerated, 8 SD, 1 PR, Median overall survival between 4.3 and 9.2 months
Nakao et al., 2011 [133]	I (6)	IT during surgery	HF10 oncolytic herpes virus	Well tolerated, 3 SD, 1 PR, 2 PD
Lutz et al., 2011 [134]	II (60)	ID	Plasmid/GVAX with chemoradiation	Well tolerated Median disease free survival 17.3 months (resected tumors)
Kubusho et al., 2012 [135]	I (7 with mutated *KRAS*)	SC	EBV/Peripheral blood lymphocytes genetically modified with an episomal EBV expressing Ras mutant	No acute EBV infection 4 SD, 6 T cell response
Hanna et al., 2012 [136]	I/Iia (9)	IT EUS- and CT-guided	Plasmid / expression of diphtheria-toxin gene	Well tolerated, 3 PR
Hecht et al., 2012 [137]	I (50)	IT EUS- and CT-guided	AdV/TNFerade with chemoradiation (5 FU)	21 mild adverse events 1 complete response, 7 PR, 12 SD, 19 PD Median OS: 10 months
Le et al., 2012 [138]	I (26)	IV (GI metastatic cancers included)	Attenuated listeria vaccine expressing (17) or not (9) mesotheline	Immune response, OS > 15 months in 10 patients
Le et al., 2013 [139]		SC	A: Ipilimumab alone B: GVAX + Ipilimumab	20% Grade 3/4 immune-relate adverse events A and B : SD OS: 3.2 vs 5.7 (NS) 1 year survival 7 Vas 27%
Hardacre et al., 2013 [140]	II (70 after tumor resection)	SC	Algenpantucel-L + gemcitabine + 5FU	12% induration at the injection site one year OS: 86%
Herman et al., 2013 [141]	III (304)	IT EUS- and CT-guided	AdV/TNFerade + Chemoradiation Vs chemoradiation	More grade 1 to 2 AE in the TNFerade + chemoradiation arm OS: 10 months, both arms
Löhr et al., 2014 [142]	II (13)	angiography	Lipofectamine/Cyto. P450 (*)	Well tolerated 4 PR, 4 PD, 5 SD OS: 9.5 months
Aguilar et al., 2015 [143]	I (24)	IT EUS- and CT-guided	AdV/HSV thymidine kinase Arm A borderline: HSK −TK + valacyclovir Arm B non resectable: HSV − TK + valacyclovir + chemoradiation	Well tolerated Median OS: 10 months in Arm A and 12 months in arm B with 25% of RECIST response.
Buscail et al., 2015 [144]	I (22)	IT EUS- guided	Complexed plasmid/CYL-02 + gemcitabine	Well tolerated, 12 SD, OS in non metastatic patients 12.6 months
Golan et al., 2015 [145]	I/IIa (15)	IT EUS- and CT-guided	IT placement of SiG12-LODER^®^ *+* gemcitabine	90% minimal AE 2 PR, 10 SD, 2 PR OS: 15 months
Le et al., 2015 [119]	II (90)	SC	GVAX + CRS 2017 (Arm A) vs GVAX alone (Arm B)	Local reactions 77%, general minor AE 53–62% OS: 6.1 months in arm A Vs 3.9 months in arm B
Noonan et al., 2016 [146]	II (73)	IV	Arm A: Reolysin + paclitaxel + carboplatin Arm B: paclitaxel + carboplatin	Well tolerated No difference I term of PFS and OS between the two arms

IT: intratumoral route; ID: intradermal route; IV: intravenous route; SC: subcutaneous route; CT: CT scan; AdV: adenoviral vector; Rv: retroviral vector; PR: partial response; SD: stable disease; PD: progressive disease; PFS: progression free survival; OS: overall survival; AE: adverse event ; RECIST: Response Evaluation Criteria In Solid Tumors; ONYX-1: E1B-55 kDa region- deleted adenovirus that selectively replicates in and lyses tumor cells with abnormalities in p53 function; *: Encapsuled genetically modified allogenic fiboblastic cells (in cellulose sulfate) that expressed cytochrome P450 enzyme transforming the Isofosfamide IV; TNFerade is an adenovector encoding human human tumor necrosis alfa with a radiation-inducible promoter. Administration was combined with chemoradiation therapy; Rexin-G: cytocidal retrovirus gene construct encoding mutant cyclin G1 gene; GVAX: allogenic pancreatic cancer cells expressing and secreting GM-CSF (plasmidic transfection); Algenpantucel-L: composed of two human pancreatic cancer cell lines genetically engineered to express αGal using a retroviral transfer; HSV-TK: herpes virus thymidine kinase that phosphorylate an antiherpetic prodrug valacyclovir becoming toxic for pancreatic cancer cells expressing the suicide gene HSV-TK; CYL-02: gene therapy product combining *SSTR2 (human somatostatin receptor subtype 2)*, *DCK (deoxycytine kinase)* and *UMK* (uridyl moniphosphate kinase) genes and polycationic polyethylenimine vector with antioncogenic and chemosensitizing (gemcitabine) activities; LODER^®^: miniature biodegradable implant (intratumoral insertion) containing and releasing within 4 months *a* siRNA drug against *Kras* (mutation G12D); CRS 2017: live-attenuated listeria monocytogene genetically ingeneered to express mesothelin; Reolysin*^®^*: pelareorep, Reovirus Serotype3-Dearing Strain.

**Table 4:** Main on-going clinical trials of gene therapy for pancreatic cancer (source ClinicalTrials.gov, available online: https://clinicaltrials.gov/).

Identification Number Date of Start Trial Stage	Phase, (Patient Number)	Route	Vector/Strategy
NCT00711997 August 2009 Completed	I/II (9)	IT CT- or US- or EUS-guided	jetPEI/DTA-H19
NCT00669734 February 2010 Active, not recruiting	I (18)	IT or SC	Lowlpox and Vaccinia virus/PANVAC-V+ PANVAC-F + GM-CSF)
NCT01088789 April 2010 Recruiting	II (72)	ID	PANC 10.05 pcDNA-1/GM-Neo and PANC 6.03 pcDNA-1 neo vaccine (allogenic pancreatic tumor cell vaccine transfected with the GM-CSF gene) +/− cyclophosphamide
NCT01191684 October 2011 Completed	I (2)	SC	Vaccinia virus/Vaccinia virus ankara vaccine expressing p53
NCT01583686 April 2012 Recruiting	I/II (136)	IV	Rv/Anti-mesothelin CAR-T cell
NCT01836432 May 2013 Active, not recruiting	III (302)	ID	Rv/Algenpantucel-L +/− chemotherapy (Folfirinox or Gemcitabine + Nab-paclitaxel)
NCT02239861 September 2014 Recruiting	I (18)	IV	Tumor-associated antigen (TAA)-specific cytotoxic T lymphocytes 5 common TAAs: NY-ESO-1, MAGEA4, PRAME, Survivin and SSX.
NCT02340117 January 2015 Recruiting	II (28)	IV	Cationic liposome/SGT-53 + Gemcitabine + Nab-Paclitaxel
NCT02416466 April 2015 Active, not recruiting	I (8)	Percutan-eous hepatic artery infusion	SIR-Spheres microspheres/Anti-CEA CAR-T cells
NCT02465983 May 2015 Active, not recruiting	I (12)	IV	CART-meso-19 T cells + Cyclophosphamide
NCT02432963 November 2015 Recruiting	I (12)	ID	Vaccinia Ankara/Modified Vaccinia Ankara vector expressing full length wild type human p53 + Pembrolizumab
NCT02806687 June 2016 Recruiting	II (100)	IT EUS-guided	JetPEI/CYL-02 + Gemcitabine
NCT02576665 July 2016 Recruiting	I (26)	IV or IT	Rv/Toca 511 + Toca FC
NCT02894944 August 2016 Recruiting	I (9)		AdV/Theragene + Chemotherapy
NCT02705196 November 2016 Not yet recruiting	I/II (26)	IT EUS- or US-guided	AdV/LOAd703 + Gemcitabine + Nab-paclitaxel

IT: intratumoral route; ID: intradermal route; IV: intravenous route ; SC: subcutaneous route ; CT: CT scan; AdV: adenoviral vector; Rv: retroviral vector; CART-meso-19 T cells: patients′ own T cells modified to express a receptor specific to the mesothelin protein and receptor specific to CD19; DTA-H19: a doubled stranded DNA plasmid that carries the gene for the diphtheria toxin A (DT-A) chain under the regulation of the H19 promoter sequence; GVAX: allogenic pancreatic cancer cells expressing and secreting GM-CSF (plasmidic transfection); Algenpantucel-L: composed of two human pancreatic cancer cell lines genetically engineered to express αGal using a retroviral transfer. HSV-TK: herpes virus thymidine kinase that phosphorylate an antiherpetic prodrug valacyclovir becoming toxic for pancreatic cancer cells expressing the suicide gene HSV-TK; ADV-TK: Adenovirus-mediated herpes simplex virus thymidine kinase gene therapy; CYL-02: gene therapy product combining *SSTR2 (human somatostatin receptor subtype 2)*, *DCK (deoxycytine kinase)* and *UMK* (uridyl moniphosphate kinase) genes and polycationic polyethylenimine vector with antioncogenic and chemosensitizing (gemcitabine) activities; LOAd703 : oncolytic adenovirus modified to include additional immune system stimulators; *PANVAC-F:* Intratumoral Recombinant Fowlpox PANVAC. PANVAC-V: Subcutaneous Recombinant Vaccinia PANVAC; SGT-53: complex of cationic liposome encapsulating a normal human wild type p53 DNA sequence in a plasmid backbone; Theragene: Replication-competent Adenovirus-mediated Double Suicide Gene Therapy (Theragene^®^, Ad5-yCD/mutTKSR39rep-ADP – Theragen Pharamceuticals Inc. San Diego, CA, USA); Toca 511: a retroviral replicating vector encoding a modified yeast cytosine deaminase (CD) gene; The CD gene converts the antifungal 5-flurocytosine (5FC) to the anticancer drug 5-FU in cells that have been infected by the Toca 511 vector; Toca FC: an extended-release formulation of flucytosine (Tocagen Company, San Diego, CA, USA).

## Abbreviations

PDAC	Pancreatic Ductal Adenocarcinoma
EUS	Endoscopic Ultrasound
CAR	Chimeric Antigen Receptors
VEGF	Vascular endothetial Growth Factor
EGF	Epidermal Growth Factor
MUC-1	Mucin 1
HSV-TK	Herpes Virus Thymidine Kinase
TNF	Tumor Necrosis Factor
CAR	Chimeric Antigen Receptor
GM-CSF	Granulocyte-Macrophage Colony-Stimulating Factor
